# Pharmacist assessment of drug-gene interactions and drug-induced phenoconversion in major depressive disorder: a case report

**DOI:** 10.1186/s12888-021-03659-4

**Published:** 2022-01-20

**Authors:** N. M. Del Toro-Pagán, A. Matos, C. Bardolia, V. Michaud, J. Turgeon, N. S. Amin

**Affiliations:** 1 Office of Translational Research and Residency Programs, Tabula Rasa HealthCare, 228 Strawbridge Drive, Moorestown, NJ 08057 USA; 2 Precision Pharmacotherapy Research & Development Institute, Tabula Rasa HealthCare, Lake Nona, FL USA; 3 Faculty of Pharmacy, Université de Montréal, Montreal, Quebec, Canada

**Keywords:** Antidepressants, Case report, CYP2C19, CYP2D6, Pharmacogenetics, Pharmacogenomics

## Abstract

**Background:**

Response to antidepressant therapy is highly variable among individuals. Pharmacogenomic (PGx) testing presents an opportunity to guide drug selection while optimizing therapy outcomes and/or decreasing the risk for toxicity.

**Case presentation:**

A patient with multiple comorbidities, including severe major depressive disorder (MDD), experienced adverse drug events and undesirable response to multiple antidepressant medications (i.e., bupropion, escitalopram, and venlafaxine). A clinical pharmacist assessed significant drug-gene, drug-drug, and drug-drug-gene interactions as well as other clinical factors to provide recommendations for antidepressant therapy optimization.

**Conclusion:**

This case highlights the importance of PGx testing and the key role of pharmacists in identifying and mitigating drug-related problems and optimizing drug therapy in patients with MDD.

**Supplementary Information:**

The online version contains supplementary material available at 10.1186/s12888-021-03659-4.

## Background

Pharmacogenomics (PGx) is the study of human genome variants that impact drug response, typically through alterations in their pharmacokinetics or pharmacodynamics [1]. Overall, genetic differences are responsible for 15 to 30% of interpatient variability in drug disposition and response; however, for certain drugs, genetic differences can be responsible for up to 95% of the drug response variability [2]. When PGx results are known, drug-gene interactions (DGIs) and drug-drug-gene interactions (DDGIs) can be identified. DGIs are interactions involving a drug and coding variation in a gene leading to altered protein function or expression such as a cytochrome P450 (CYP) isoenzyme (e.g., sertraline and CYP2C19 poor metabolizer phenotype [PM]), a receptor (e.g.*,* morphine and μ-opioid receptor) or transporter (e.g., simvastatin and SLCO1B1 decreased function). A DDGI results from the superimposition of a drug-drug interaction (DDI) on a DGI, often causing phenoconversion [3]. Phenoconversion is defined as the ability of intrinsic or extrinsic factors, such as DDIs, to modify a genotype-predicted phenotypic expression [4]. For example, an individual with a CYP2D6 *1/*1 genotype predicted to exhibit a CYP2D6 normal metabolizer (NM) status is converted into a poor metabolizer (PM) while taking quinidine, a potent CYP2D6 inhibitor. Hence, the disposition of CYP2D6 substrates such as risperidone would be impaired in NM individuals during quinidine treatment, possibly increasing the risk of risperidone-related toxicity.

Genotyping can be done preemptively or reactively. Preemptive PGx testing allows for the availability of test results prior to the selection of a medication for which PGx clinical guidelines are available, thus enabling clinicians to better tailor drug selection [5]. Reactive PGx testing is generally completed under specific circumstances; for example, a patient experiencing pharmacotherapy failure or adverse drug events (ADEs) [6]. Organizations such as the Clinical Pharmacogenetics Implementation Consortium (CPIC) and the Dutch Pharmacogenetics Working Group (DPWG) facilitate the use of PGx in clinical practice and have developed clinical guidelines to ease drug and dose selection based on current evidence [7].

Recently, the therapeutic area of psychiatry has made efforts for achieving more precise treatments by considering interindividual differences in relevant genes for drug action [8]. Antidepressant response and efficacy vary among patients with major depressive disorder (MDD) [9]. Patients often experience long periods of poor management of depression symptoms and/or ADEs until the right medication and dose is established [10]. The initial antidepressant treatment response rate is about 50%, while depression remission rate are around 37% [11]. Interpatient variability in antidepressant drug treatment response and efficacy can be attributed to environmental, physiological, and psychological factors, comorbidities, and genetic variability [12]. Evidence indicates that genes encoding for drug metabolizing enzymes that can impact pharmacokinetic parameters (e.g.*, CYP2C19, CYP2D6)* may have a significant impact in the response and efficacy of such medications. In particular, CYP2C19 and CYP2D6 drug metabolizing enzymes are the primary focus for PGx in psychiatry because they predominantly contribute to the phase I metabolism of most currently available antidepressants, while the CYP2D6 enzyme contributes to the metabolism of many antipsychotics [13]. *CYP2C19* and *CYP2D6* genetic polymorphisms can affect the pharmacokinetic parameters (i.e.*,* clearance, half-life, and plasma concentrations) of most antidepressants, thus increasing the risk for ADEs and impacting therapeutic outcomes [14]. Therefore, PGx testing can help predict antidepressant tolerability and response [15], potentially enabling a more safe, effective, and cost-effective treatment [16]. Although integrating PGx testing into the management of MDD has been implemented at some institutions, the widespread adoption of this practice has still to overcome many barriers such as, as insufficient evidence generation, data sharing and slow uptake of genomic information [17].

The case described herein demonstrates a unique approach leading to optimized antidepressant therapy in a patient. This approach consisted of a clinical pharmacist performing a PGx-informed assessment assisted by the proprietary clinical decision support system (CDSS), MedWise™ which has been described elsewhere [18]. This CDSS incorporates PGx results in combination with the medication regimen to support the clinician on the identification of clinically significant DDIs, DGIs, DDGIs, and drug-induced phenoconversion. Other clinical factors are also considered to provide recommendations to optimize antidepressant therapy.

## Case presentation

A 76-year-old female presented with MDD with inadequate response to antidepressants, post-traumatic stress disorder, obsessive compulsive disorder, generalized anxiety disorder, pseudobulbar affect, insomnia, restless leg syndrome, constipation, back pain, gastroesophageal reflux disease, hyperlipidemia, and glaucoma. Smoking status was not disclosed. Hepatic, kidney, and thyroid function laboratory values were all within normal limits (e.g., albumin, AST, ALT, creatinine, BUN, total protein, globulin, alkaline phosphatase, and bilirubin total, thyroid-stimulating hormone). The medications prescribed by the primary care provider (PCP) to manage her various medical conditions are listed in Table 1.

**Table 1:** Patient’s Medication List

Medical condition	Medication	Dose	Frequency
**MDD**	Quetiapine IR	100 mg	Twice a day
**PTSD**	Quetiapine XR	200 mg	Daily
**OCD**	Risperidone	0.5 mg	Twice a day
**GAD**	Sertraline	100 mg	Daily
	Duloxetine DR	30 mg	Daily
**Pseudobulbar effect**	Dextromethorphan/quinidine	20/10 mg	Every 12 h
**Insomnia**	Trazodone	100 mg	Daily at bedtime
**Restless leg syndrome**	Gabapentin	100 mg	Daily at bedtime
**Constipation**	Magnesium hydroxide suspension	400 mg/5 mL	Daily at bedtime
	Polyethylene glycol 3350	17 g	Daily as needed
**Back pain**	Acetaminophen	500 mg	Every 4 h as needed
**GERD**	Pantoprazole DR	40 mg	Daily
	Famotidine	20 mg	Twice a day
**Hyperlipidemia**	Rosuvastatin	40 mg	Daily
**Glaucoma**	Timolol 0.5%	1 drop in each eye	Daily

Abbreviations: *DR* Delayed Release, *GAD* Generalized Anxiety Disorder, *GERD* Gastroesophageal Reflux Disorder, *IR* Immediate Release, *XR* Extended Release, *MDD* Major Depression Disorder, *OCD* Obsessive Compulsive Disorder, *PTSD* Post-Traumatic Stress Disorder

The patient’s chief complaint was uncontrolled depression despite multiple attempts with various antidepressants. These medications include bupropion, escitalopram, and venlafaxine which all resulted in the patient experiencing ADEs and/or inadequate depression control. In brief and based on medical history, the patient was initially started on bupropion which was discontinued as she experienced uncontrolled shaking. Escitalopram was then used for 2 months and was discontinued due to an unknown reaction. Subsequently, venlafaxine was used for 3 months without favorable outcomes. This led to the introduction of other medications, such as: sertraline, risperidone and duloxetine. Additionally, this patient was also treated with quetiapine, although initiation date of treatment was unknown. The timeline for her antidepressant trials and other concomitant medications, including prescription and over the counter, is depicted in Table 2.

**Table 2:**
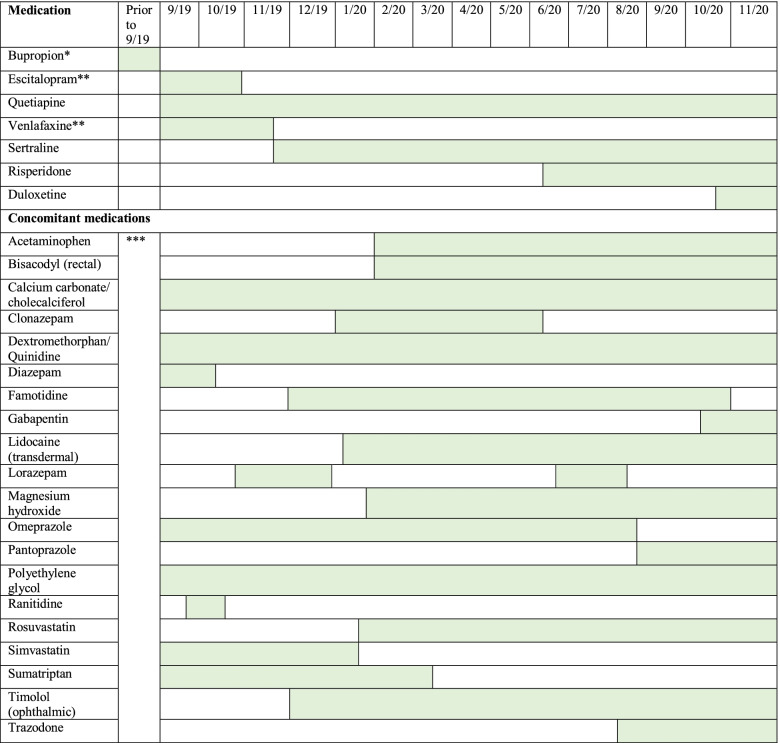
PCP care – Reported antidepressant trials along with concomitant medications^†^

Abbreviations: *PCP* Primary Care Provider, *ADE* Adverse Drug Event

^*^Discontinued past medication trial because of ADEs

^**^Discontinued past medication trial for unknown reasons

^***^Unknown status

^†^Shaded area represents the period of time the patient was on each medication

This participant had recently enrolled in the Program of All-inclusive Care for the Elderly (PACE). Within the PACE model, pharmacists and other healthcare practitioners collaborate to identify and mitigate medication-related problems [19]. PGx testing is one of the initiatives utilized to further improve the care of PACE participants. Being newly introduced to the management of this patient, a clinical pharmacist recommended a PGx test to optimize MDD management; this recommendation was accepted by the PCP. A DNA sample was collected via buccal swab and analyzed by a genetic laboratory (CQuentia, Memphis, TN; Genetic Response Report) and the clinical pharmacist was consulted to interpret relevant pharmacogenomic results. The patient was identified as a CYP2C19 IM, with a CYP2C19*2|*17 genotype, and as a CYP2D6 IM, with a CYP2D6*1|*4 genotype.

Although the clinical pharmacist assessed the complete drug regimen, only recommendations relevant to antidepressant and antipsychotic therapies will be discussed in this case report. It is also worth acknowledging that although the patient’s chief complaint was uncontrolled depression and focus was on the MDD diagnosis, there are common symptoms with regards to her present psychiatric comorbidities. DGIs were considered relevant for the metabolism of duloxetine and risperidone (CYP2D6), and for the disposition of sertraline (CYP2C19). The CYP2D6 IM phenotype is associated with reduced enzyme activity and decreased clearance of CYP2D6 substrates. Hence, the risk of toxicity is increased for risperidone and to a lesser extent for duloxetine; it should be noted that the major metabolic pathway for duloxetine is through CYP1A2 (70%), while the contribution of 2D6 is limited to 30%. Similarly, a CYP2C19 IM has reduced enzyme activity, which results in decreased sertraline clearance and increased risk of toxicity.

The clinical pharmacist also identified three clinically significant DDIs (Table 3). Quinidine, a potent CYP2D6 inhibitor, is expected to inhibit the metabolism of risperidone and duloxetine. Such inhibition occurs regardless of the time of administration of the drugs as this interaction is mechanistically a non-competitive inhibition. The CDSS, which is based on algorithms and several pharmacological factors, was used to determine the presence of drug-induced phenoconversion (patent: WO 2019/089725). Quinidine inhibition of CYP2D6 resulted in phenoconversion whereby this patient’s phenotype is converted from a CYP2D6 IM to a PM phenotype. When this interaction occurs, the plasma concentrations of risperidone and duloxetine are likely to be higher than predicted from the genotypic results alone. Lastly, DDIs were also identified by the CDSS on the CYP3A4 metabolic pathway; risperidone and quinidine are drugs with stronger affinity for the CYP3A4 enzyme than quetiapine. Consequently, these drugs are expected to competitively inhibit the metabolism of quetiapine when co-administered. When this interaction occurs, the plasma concentrations of quetiapine are likely to be significantly higher than predicted (CYP3A4 contributes to 75% of the total clearance of quetiapine), increasing the risk for toxicity. A similar mechanism of DDI occurs between sertraline —a CYP3A4 substrate with low affinity— risperidone, and quetiapine. CYP3A4 is responsible of 25% of the total clearance of sertraline, leading to moderately clinically significant changes in plasma concentrations of sertraline (if only this route of elimination is affected).

**Table 3:**
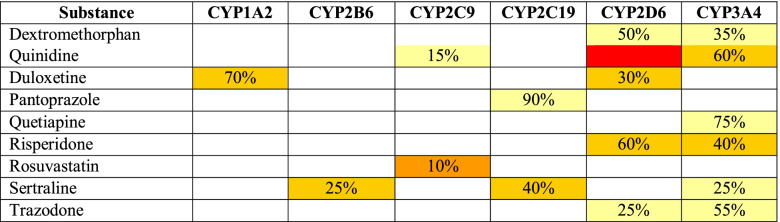
Summary of Affinity and CYP450 Metabolic Pathways [18]^ǂ^

MedWise™ depicts the various degrees of binding affinity of a substrate for a specific enzyme using different colors (e.g., light yellow for weak affinity and dark yellow for moderate affinity. The percentages listed correspond to the use of the metabolic pathway for the substrate

Legend:

Abbreviations: *CYP* Cytochrome P450

^ǂ^Only CYP-metabolized oral drugs are displayed

When performing the assessment and making recommendations, the clinical pharmacist considered several factors including previous unsuccessful medication trials, PGx test results and concomitant medications. Quetiapine has a low affinity for the dopamine 2 receptor, which is required for antipsychotic efficacy, therefore higher doses may be required for clinical effects for mood disorders and agitation. Additionally, quetiapine has mixed results for the treatment of dementia psychosis and agitation [20]. Given this information and the DDI impacting the metabolism of quetiapine at CYP3A4, it was recommended that the PCP taper off the quetiapine while simultaneously optimizing the risperidone dose and frequency (targeting the lowest effective dose). Continued monitoring (e.g., EKG, palpitations) for risperidone was also suggested due to an increased risk of QTc prolongation caused by the presence of a DGI and a DDI at CYP2D6, and by the combination of quinidine [21], risperidone [22], rosuvastatin [23], quetiapine [24], and pantoprazole [25], which have all been associated with drug-induced QTc prolongation. Furthermore, it was recommended to optimize antidepressant therapy dosing based on clinical response and presence of ADEs.

Over the next 8 weeks the aforementioned recommendations were accepted by the PCP, who before implementing had a thorough discussion with the patient’s psychiatrist to account for other non-PGx factors. Tapering of the quetiapine dose was attempted and risperidone did not require a further increase in dose. Antidepressant therapy was optimized by increasing the duloxetine dose from 30 mg to 60 mg daily, the patient was monitored closely during dose escalation. It was determined that the dose of sertraline would be re-assessed based upon therapeutic response to these changes. The PCP reported that the patient was experiencing less anxiety and better control of depression as frequent monitoring was continued.

## Discussion and conclusions

Uncontrolled depression is a major health issue that has significant individual and social-economic impact. In a systematic review conducted by Mrazek et al., it was shown that patients with uncontrolled depression have a 15% likelihood of suicide ideation compared to 6% for patients who respond appropriately to antidepressant therapy [26]. Hampton et al. estimated that each year over 25,000 patients in the United States present to the emergency department due to antidepressant-related ADEs [27]. Corponi et al. estimate that only around one third of patients with MDD, bipolar disorder, and/or schizophrenia are compliant with their medications and are able to achieve full and stable remission [28]. Patients who do not have an adequate response to their antidepressant regimens are expected to have higher healthcare-related costs (about $10,000 more yearly) than patients who do respond appropriately [26].

PGx test results combined with appropriate CDSS and clinical pharmacist interventions can help patients achieve depression remission quicker, decrease the number of ADEs, and potentially decrease healthcare costs related to trial-and-error approaches [29]. There are still conflicting results regarding the clinical utility and cost-effectiveness of implementing PGx testing into routine clinical care despite PGx being a promising tool to guide treatment selection for patients with MDD [15]. This is explained in part by the fact that antidepressant plasma concentrations seem to poorly correlate with clinical efficacy [30]. Indeed, plasma concentrations remain an indirect estimation of drug concentrations in the effect-compartment (central nervous system), and several factors could explain this poor relationship such as distribution to the central nervous system limited by influx and efflux transporters expressed at the blood-brain barrier, inability to measure drug concentrations next to binding site on target receptors, and disease states [31]. However, dose adjustments based on PGx results for drug-metabolizing enzymes appear to provide more reliable information on the risk for ADEs [30]. Polymorphisms in the serotonin transporter and the tryptophan hydroxylase can also impact antidepressant response; however, insufficient data exists to support testing on these genes to modify antidepressant regimen [30, 32].

CPIC has developed evidence-based guidelines for sertraline therapy (Table S1) in patients who have readily available PGx information [33], while DPWG provides guidance for duloxetine [34], ,risperidone [35], and sertraline (Table S2) [36]. Around 40% of the total clearance of sertraline is mediated by CYP2C19 (Table 3), and its plasma concentrations are expected to be increased in CYP2C19 IMs. Additionally, a DDI was identified at CYP3A4 which could further increase the concentrations of sertraline (CYP2C19 DGI plus CYP3A4 DDI may decrease up to 65% of the total clearance of sertraline). Nevertheless, for IMs, both CPIC and DPWG guidelines recommend initiating sertraline therapy at the recommended starting dose [33, 36]. For the maintenance dose of sertraline, monitoring of ADEs is warranted and adjustments should be guided by therapeutic response (i.e.*,* efficacy and toxicity), as mean steady-state plasma levels could increase with time. In this case, a close monitoring of potential side effects with sertraline was recommended due to the DGI combined with DDI. DPWG does not provide dosing recommendations for the duloxetine and *CYP2D6* drug-gene pair since genetic variations have a limited effect on the plasma concentration of this medication (CYP2D6 mediated partial metabolic clearance is 30%, while 70% is via CYP1A2) [34]. Due to the occurrence of phenoconversion at the CYP2D6 metabolic pathway, resulting in a CYP2D6 PM status, DPWG recommends reducing risperidone dose to 2/3 of the standard dose to decrease the risk of central nervous system-related ADEs [35]. Additionally, this patient was concomitantly taking four QTc-prolonging medications: quinidine [21], risperidone [22], rosuvastatin [23], quetiapine [24], and pantoprazole [25]. DGIs and DDIs must be considered when evaluating QTc-prolongation risk. DGIs and DDIs can impact drug concentrations throughout the body, including intracellular concentrations in the heart — where the binding site to the rapid component of the delayed rectifier potassium channel (I_Kr_ or hERG) is located — which can further increase the risk of QTc prolongation [37, 38]. QTc prolongation is clinically relevant due to the risk of deterioration into Torsade de Pointes, ventricular fibrillation, and sudden death which can potentially be prevented with early recognition of risk factors [37, 39].

Lastly, PGx results can also help explain why this patient previously experienced undesirable effects with bupropion, escitalopram, and venlafaxine [32]. Bupropion is significantly cleared by CYP2B6 (50%), for which we did not obtain genetic results. Bupropion is also a potent CYP2D6 inhibitor which likely impacted the metabolism of other concomitant medications at this time. This could have misled the causal clinical observations that side-effects were related to bupropion — not to concomitant treatments — leading to its discontinuation. CYP2C19 is responsible for 50% of escitalopram metabolic clearance while CYP2D6 contributes about 10%. This patient is a CYP2C19 IM and phenotypically a CYP2D6 PM (due to phenoconversion). These conditions likely impaired the clearance of escitalopram, possibly leading to increased escitalopram plasma concentrations that could have explained the need for treatment discontinuation. Venlafaxine is metabolized by CYP2C19 (30%) and CYP2D6 (50%). Phenoconversion at CYP2D6 and the drug-gene interactions on both CYP450 isoforms likely caused increased venlafaxine concentrations (potential loss of 80% of the total clearance) which may have contributed towards undesirable ADEs.

A limitation of using PGx results to optimize antidepressant regimens is that not all health care professionals (e.g., physicians, pharmacists) are adequately trained to interpret these results. Furthermore, other factors such as, sex, smoking status, and comorbidities, can also contribute to interpatient variability of antidepressant response. In its current state, our unique approach does not consider other factors that can cause phenoconversion of the CYP450 enzymes, such as chronic inflammation [40]. We are studying closely this phenomenon which would be considered in future assessments [41]. Additionally, dietary habits that could affect drug response were not assessed.

*CYP2C19* and *CYP2D6* genetic variations and concomitant DDIs that lead to phenoconversion can significantly affect antidepressant tolerability and response. As illustrated by this patient case, some individuals are more susceptible to ADEs and may not respond appropriately to specific antidepressant therapy. Uncontrolled depression can result in noncompliance and potentially serious ADEs, such as suicidal ideation and behaviors. Implementing PGx testing into clinical practice with a clinical pharmacist that can identify and mitigate drug-related problems and optimize therapy can decrease ADEs, decrease polypharmacy, help patients achieve depression remission quicker, and potentially reduce healthcare-related costs. This case highlights the importance and clinical utility of PGx information and DGI data. Furthermore, it exemplifies pharmacist’s role in helping other healthcare practitioners select the most precise antidepressant therapy, especially under conditions of genotype-to-phenotype mismatches due to concomitant drug administration.

## Supplementary Information

**Additional file 1: Table S1.** CPIC Recommendations to Guide Sertraline Therapy Considering CYP2C19 Phenotype [33].

**Additional file 2: Table S2.** DPWG Recommendations to Guide Duloxetine and Risperidone Therapy Considering CYP2D6 Phenotype, and Sertraline Therapy Considering CYP2C19 Phenotype [34–36].

## Data Availability

All data generated or analyzed during this study are included in this published article and its supplementary information files.

## Abbreviations

ADE: Adverse Drug Event
CPIC: Clinical Pharmacogenetics Implementation Consortium
CYP: Cytochrome P450
DDI: Drug-Drug Interaction
DDGI: Drug-Drug-Gene Interaction
DGI: Drug-Gene Interaction
DPWG: Dutch Pharmacogenetics Working Group
DR: Delayed Release
GAD: Generalized Anxiety Disorder
GERD: Gastro-Esophageal Reflux Disease
IM: Intermediate Metabolizer
IR: Immediate Release
MDD: Major Depressive Disorder
NM: Normal Metabolizer
OCD: Obsessive Compulsive Disorder
PCP: Primary Care Provider
PGx: Pharmacogenomics
PM: Poor Metabolizer
PTSD: Post-Traumatic Stress Disorder
SSRI: Selective Serotonin Reuptake Inhibitor
XR: Extended Release
