# Supplementary material for: Pharmacist assessment of drug-gene interactions and drug-induced phenoconversion in major depressive disorder: a case report
Source: BMC Psychiatry. 2022 Jan 20;22:46. doi: 10.1186/s12888-021-03659-4 (PMC8772164; doi:10.1186/s12888-021-03659-4)
Supplement: Supplementary file 1 — Additional file 1: Table S1. CPIC Recommendations to Guide Sertraline Therapy Considering CYP2C19 Phenotype [33]. [file 12888_2021_3659_MOESM1_ESM.docx]

# Supplementary Material:

## **Table S1: CPIC Recommendations to Guide Sertraline Therapy Considering CYP2C19 Phenotype(33)**

| **CYP2C19 Phenotype** | **Implications and Clinical Interpretation** | **Recommendations** |
| --- | --- | --- |
| Ultra-Rapid or Rapid Metabolizer | Increased metabolism when compared to normal metabolizers. | Initiate therapy with recommended starting dose. If patient does not respond to recommended maintenance dosing, consider alternative drug not predominantly metabolized by CYP2C19. |
| Intermediate Metabolizer | Reduced metabolism when compared to normal metabolizers. | Continue to follow label recommended dosing. |
| Poor Metabolizer | Greatly reduced metabolism when compared to normal metabolizers. Higher plasma concentrations may increase the probability of side effects. | Consider a 50% reduction of recommended starting dose and titrate to response or select alternative drug not predominantly metabolized by CYP2C19. |

Abbreviations: CPIC: Clinical Pharmacogenetics Implementation Consortium; CYP: Cytochrome P450
