# Supplementary material for: Pharmacist assessment of drug-gene interactions and drug-induced phenoconversion in major depressive disorder: a case report
Source: BMC Psychiatry. 2022 Jan 20;22:46. doi: 10.1186/s12888-021-03659-4 (PMC8772164; doi:10.1186/s12888-021-03659-4)
Supplement: Supplementary file 2 — Additional file 2: Table S2. DPWG Recommendations to Guide Duloxetine and Risperidone Therapy Considering CYP2D6 Phenotype, and Sertraline Therapy Considering CYP2C19 Phenotype [34–36]. [file 12888_2021_3659_MOESM2_ESM.docx]

## **Table S2: DPWG Recommendations to Guide Duloxetine and Risperidone Therapy Considering CYP2D6 Phenotype, and Sertraline Therapy Considering CYP2C19 Phenotype(34-36)**

| **Phenotype** | **Implications and Clinical Interpretation** | **Recommendations** |
| --- | --- | --- |
| **Duloxetine – CYP2D6** | | |
| Ultra-Rapid, Intermediate, or Poor Metabolizer | Although plasma concentrations of duloxetine may be affected, a typical response is expected. | There are currently no dosing recommendations for duloxetine based on CYP2D6 genotype. |
| **Risperidone – CYP2D6** | | |
| Ultra-Rapid Metabolizer | The percentage of patients with therapy failure increases from 16% to 37%. The gene variation leads to a high ratio of the active metabolite (9-hydroxyrisperidone (paliperidone)) compared to risperidone, which crosses the blood-brain barrier more effectively. | Choose an alternative or titrate the dose according to the maximum dose for the active metabolite (paliperidone) (oral 12 mg/day for adults and children from 15 years of age weighing at least 51 kg and 6 mg/day for children from 15 years of age weighing less than 51 kg; intramuscular 75 mg per 2 weeks). |
| Intermediate Metabolizer | There is little evidence to support an increase in side effects caused by the gene variation. The gene variation may lead to a decrease in the required maintenance dose. However, as the effect on the dose is smaller than that of the normal biological variation, action is not useful. | Continue to follow label recommended dosing. |
| Poor Metabolizer | The percentage of patients with therapy failure increased from 16% to 26%. The gene variation increases the plasma concentration of risperidone plus the active metabolite and increases the proportion of risperidone in this ratio, which is more effective at crossing the blood-brain barrier. | Use 67% of the standard dose. If problematic side effects originating in the central nervous system occur despite this reduced dose, then reduce the dose further to 50% of the standard dose. |
| **Sertraline – CYP2C19** | | |
| Ultra-Rapid Metabolizer | The gene variation has a negligible effect on the plasma concentration of sertraline. Moreover, no significant effect on response and side effects has been found. | Continue to follow label recommended dosing. |
| Intermediate Metabolizer | The gene variation has a minor effect on the sertraline plasma concentration. No effect on side effects has been found. | Continue to follow label recommended dosing. |
| Poor Metabolizer | The risk of side effects is increased. The gene variation leads to increased plasma concentrations of sertraline. | Do not give doses exceeding 75 mg/day. Guide the dose by response and side effects and/or sertraline plasma concentration. |

Abbreviations: CYP: Cytochrome P450; DPWG: Dutch Pharmacogenetics Working Group
